# The BVI ISOPURE^®^ 123 intraocular lens: a new hydrophobic preloaded extended monofocal IOL with intermediate vision correction

**DOI:** 10.3389/fopht.2023.1330335

**Published:** 2024-02-01

**Authors:** Ejaz Ansari

**Affiliations:** ^1^ Eye Ear and Mouth Unit, Maidstone & Tunbridge Wells Hospitals, Kent, United Kingdom; ^2^ Institute of Medical Sciences, Canterbury Christ Church University, Kent, United Kingdom

**Keywords:** extended depth of field/focus, intraocular lens, distance vision, intermediate vision, dysphotopsias

## Abstract

The BVI ISOPURE^®^ intraocular lens (IOL) (PhysIOL, Liege, Belgium) is a fully refractive, aspherical, monofocal lens based on isofocal technology. Isofocal technology provides cataract patients distance vision quality combined with improved unaided intermediate vision by accentuating the extended depth of field/focus (EDOF) effect whilst inducing minimal photic phenomena.

**Methods:**

PubMed, Web of Science, Scopus, and Google Scholar searches were conducted for published research articles featuring the ISOPURE 123 IOL.

**Results:**

Excellent uncorrected and corrected binocular distance visual acuity of at least 20/25 can be achieved; uncorrected binocular intermediate vision of 20/25 or better in 81% and 50% at 80 cm and 66 cm, respectively, and 42% binocular near vision of 20/40 or better can be achieved. The defocus curve showed good visual acuity at far and intermediate distances with a depth of focus value of 1.50 D. Photic phenomena are minimal compared to other EDOF IOLs. Excellent contrast sensitivity was maintained compared to a standard monofocal IOL.

**Conclusion:**

Studies show that this isofocal optic design IOL provides excellent visual performance for far vision and functional intermediate vision with an increased range of vision with few photic phenomena. This lens is an effective option for providing functional intermediate vision and correcting aphakia.

## Introduction

There are growing expectations amongst patients to be free of glasses following cataract surgery. There is now a vast array of intraocular lenses that deliver uncorrected visual acuity at various distances with fewer dysphotopsias than other types of IOL. “Extended depth-of-focus (EDOF)” IOLs create a single elongated focal point that extends the range of vision (1). In addition, fewer photic phenomena are observed compared to multifocal IOLs and trifocal IOLs (2).

This mini-review should be read with reference to the “Perspectives” section of this book (Presbyopia-correcting intraocular lenses). The reader will then understand the reasons for the parentheses around “EDOF”; a more correct term being “increased range of focus” (IROF) IOL.

ISOPURE^®^ (PhysIOL, Liege, Belgium) is a yellow hydrophobic aspheric non-diffractive IOL. Its EDOF properties are based on a polynomial surface design across the full optic providing a high amount of negative spherical aberrations, smoothly increasing from the centre to the periphery of the optic. The modification of spherical aberrations is termed isofocal technology. Isofocal optic design IOL provides excellent visual performance for far vision and functional intermediate vision with an increased range of vision.

## Methodology

PubMed, Web of Science, Scopus, and Google Scholar searches were conducted for published research articles featuring the ISOPURE 123 IOL. A total of 23 articles were reviewed with the time interval being from 2019 to 2023. Published articles rather than conference abstracts were included as per the requirements of the publisher.

## Summary of literature on visual performance

In patients undergoing same-day bilateral cataract surgery, the refractive aim being mini-monovision, ISOPURE^®^ IOL provided an extended range of functional vision, up to 63 cm, resulting in useful uncorrected near vision, good uncorrected intermediate vision, and excellent uncorrected distance vision. Subjective patient satisfaction for spectacle independence and fewer dysphotopsias was high (3).

Various studies have demonstrated the increased range of focus achieved by ISOPURE^®^. In non-comparative study, ISOPURE^®^ IOL provided excellent visual performance for far vision and functional intermediate vision with an increased range of vision and good tolerance of residual refractive cylinder (4, 5). In another non-comparative study, ISOPURE^®^ IOL, based on greater depth of focus than aspheric monofocal IOLs, offered a good option for distance and intermediate vision with fewer optical aberrations and dysphotopsias (6). In a randomised controlled trial comparing ISOPURE^®^ IOL with a standard monofocal aspheric IOL, both IOLs provided excellent visual acuity and contrast sensitivity for far vision with a similar frequency of photic phenomena, but, in addition, the ISOPURE IOL improved unaided intermediate vision performance (7).

In a study comparing different IROF IOLs with different mechanisms of action, there were no statistically significant differences in terms of frequency of photopic contrast sensitivity, halo, or glare perception. In patients without ocular comorbidities, the Eyhance ICB00 IOL (Johnson & Johnson Vision, Wokingham, UK), the Vivinex Impress IOL (Alcon Eyecare UK Ltd, Camberley, UK), and the ISOPURE^®^ IOL, even though based on different optical properties, provided similar results in terms of visual acuity, contrast sensitivity, and intraocular aberrations, with no influence of dysphotopsias (8).

In another study comparing IROF IOLS with different optical properties, each IOL provided good distance vision without creating spontaneous complaints of photic phenomena. These IROF IOLs, including ISOPURE^®^, are efficient for patients wanting spectacle independence with fewer associated dysphotopsias (9).

Another advantage of the isofocal optic design is that the modification of the surfaces of the isofocal lens does not have a negative impact on the refraction obtained by autorefraction compared to a standard monofocal intraocular lens (10).

Specific data on the occurrence of photic phenomena and optic aberrations showed that in one study, 63% had no dysphotopsias, 16% experienced glare, 16% starburst, and 5% haloes (3). When ISOPURE 123 was compared with a standard monofocal MICROPURE 123 IOL, the average size and intensity of halo (from pre-op 18.31 to 27.32) and glare (from pre-op 10.34 to 18.76) phenomena were similar between the two groups (7).

In a study comparing aberrometry with a monofocal IOL (Tecnis PCB00) (6), the coma, spherical aberration, and higher-order aberrations were similar between groups (*p*=0.45). Some differences were noted in the internal spherical aberration Z (4, 0), more negative in the ISOPURE IOL but not statistically significant (*p*=0.024).

When comparing optical quality with two other extended monofocal IOLs (Tecnis Eyhance ICB00, the Hoya Vivinex Impress XY1-EM), the point spread function (PSF) (0.193 ± 0.042), intraocular low order aberration (LOA) (0.238 ± 0.110), intraocular high order aberration (HOA) (0.166 ± 0.047), intraocular spherical aberration (SA) (0.060 ± 0.035), and ocular scatter index (OSI) (1.287 ± 0.485) were similar between the groups (*p* = 0.184, *p* = 0.108, *p* = 0.092, *p* = 0.147 and *p* = 0.544, respectively) (8). Also, at 3-months post-operatively, results of Items 17 and 38 of the NEI-RQL-42 questionnaire were satisfactory in all three groups, with complete absence of glare or halo perception.

## Results

**Figure 1:**
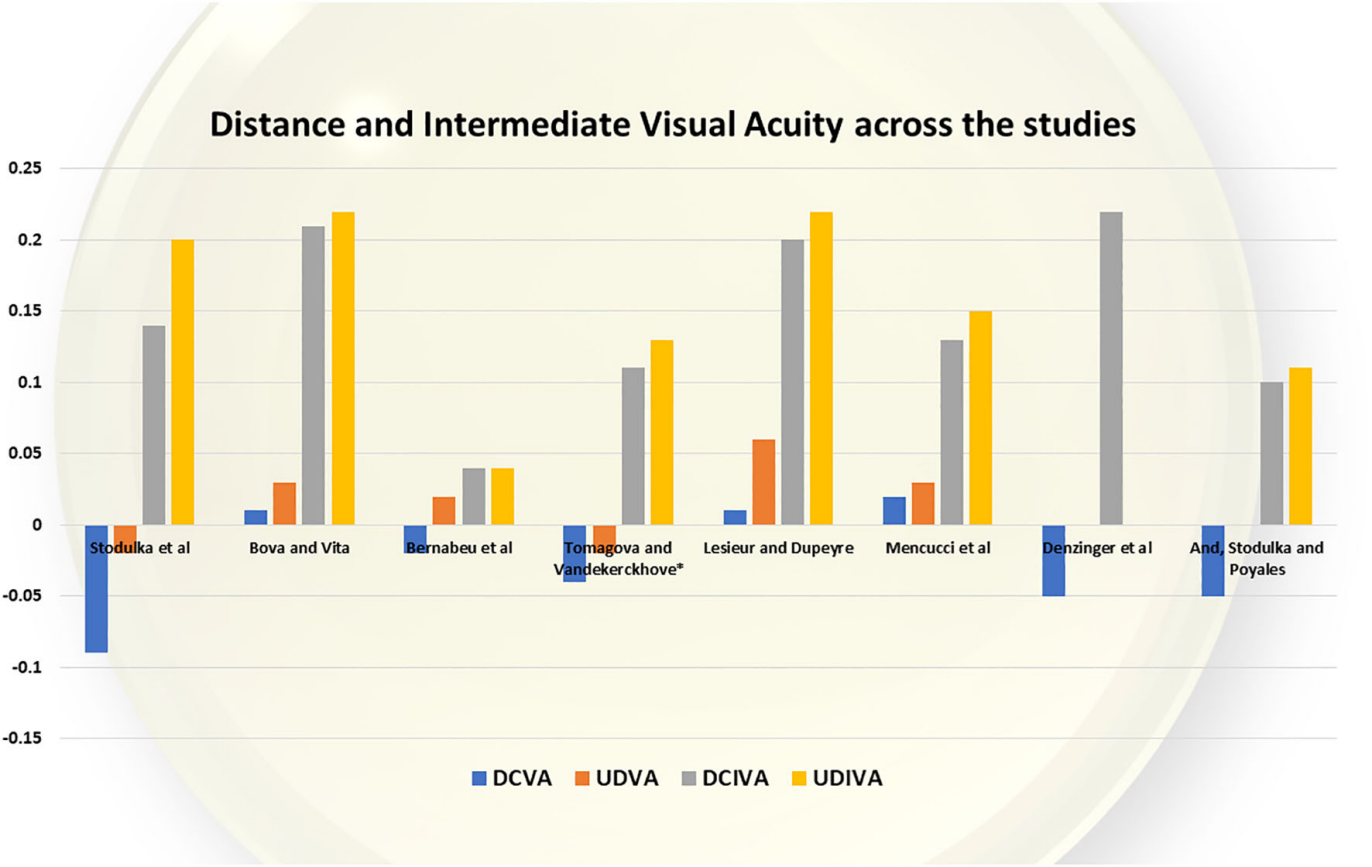
Distance and intermediate vision achieved.

**Figure 2:**
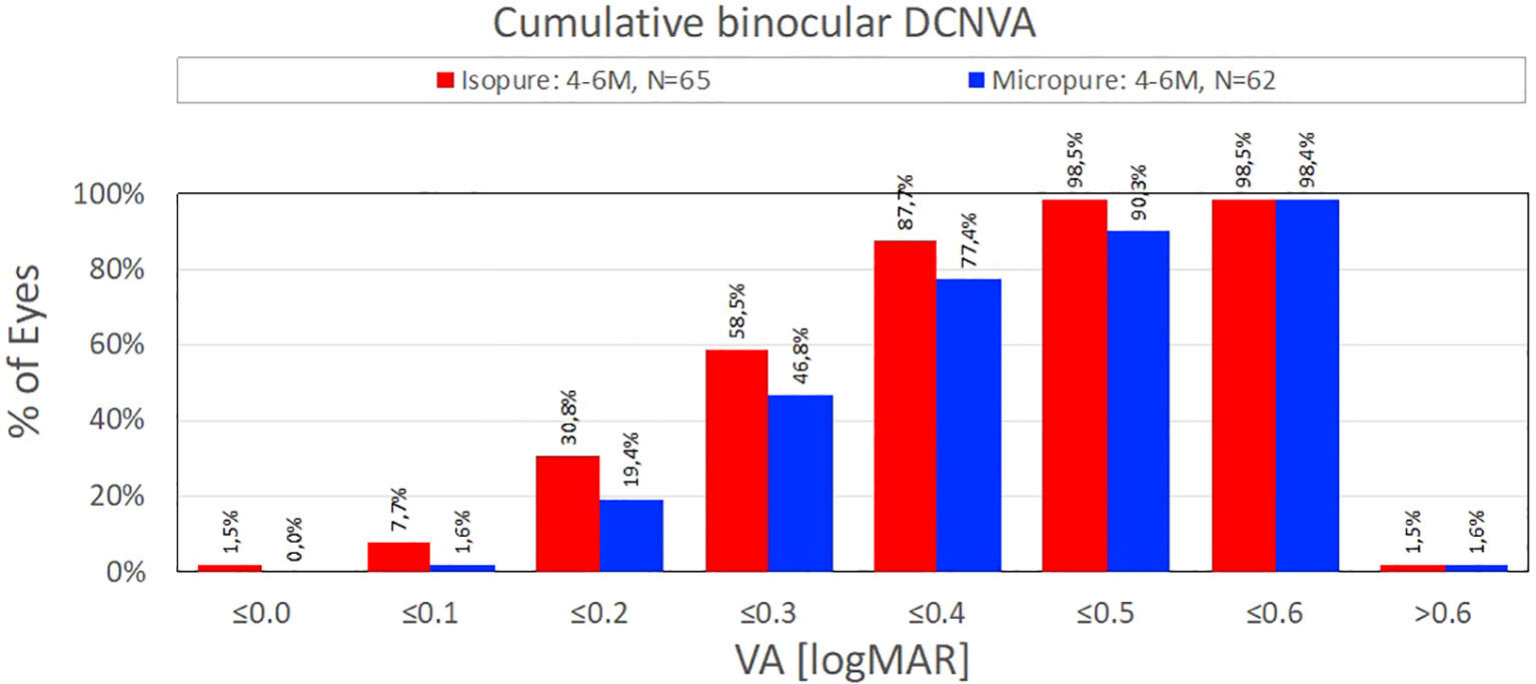
Near vision achieved.

The results are summarised in **Table 1** and **Figures 1** and **2**. Together, these data show the number of cases analysed in each study, uncorrected and best corrected distance, intermediate and near visual acuity, and a comparison of defocus curves.

**Table 1:** Clinical studies reporting outcomes of patients implanted with the Isopure 1.2.3. intraocular lens.

Authors (year)	Eyes (patients)	Follow-up (months)	Comparator *Study design*	Conclusions
**Stodulka and Slovak (** 5) **(2021)**	36 (18)	6	None *Prospective*	Isopure IOL provides excellent CDVA and DCIVA along with high contrast sensitivity and good tolerance of residual refractive cylinder.
**Bova and Vita (** 6) **(2022)**	42 (21)	12	Tecnis PCB00 IOL *Retrospective* *non-randomized*	Isopure IOL may offer a good option for the distance and intermediate vision without increasing optical aberrations and any photic phenomena.
**Bernabeu-Arias et al. (** 4) **(2023)**	183 (109)	4	None *Retrosprective - prospective*	Isopure IOL provides excellent visual performance for far vision and functional intermediate vision with an extended range of vision. This lens is an effective option for providing functional intermediate vision and correcting aphakia.
**Tomagova et al. (** 3 )* **(2023)**	124 (62)	1-2	None *Retrospective*	Isopure IOL provided an extended range of functional vision (up to 63 cm) resulting in useful UNVA, good UIVA, and excellent UDVA. Subjective patient satisfaction in terms of spectacle independence and photic phenomena was high.
**Lesieur and Dupreye (** 9) **(2023)**	22 (22)	3	Synthesis+ IOL Lucidis IOL *Retrospective*	Lucidis IOL showed more efficient distance, low contrast, and near vision than Isopure and Synthesis+ IOLs. Synthesis+ IOL seems to be more efficient in intermediate vision than other groups.
**Mencucci et al. (2023)** (8)	12 (12, 12)	3	Retrospective non randomized (3 groups, 12 patients)	In conclusion, Eyhance, Impress and Isopure enhanced monofocal IOLs—even though based on different optical profiles—showed similar results in terms of visual outcomes, aberrometry and photic phenomena perception.
**Ang, Stodulka, Poyales** **(2023) (** 7)	130 (65)	4-6	Micropure 1.2.3. IOL *Prospective* *randomized*	Both IOLs provide excellent visual acuity and contrast sensitivity for far vision with similar photic phenomena, and the Isopure IOL improved unaided intermediate vision performance.

*bilateral implantation with mini-monovision (-0.50D); IOL, intraocular lens.

**Table 1** summarises the results from the different studies, which include comparisons with other IOLs in this category.

**Figure 1** summarises the distance and intermediate visual acuities across the studies for ISOPURE 123 IOL.

**Figure 3:**
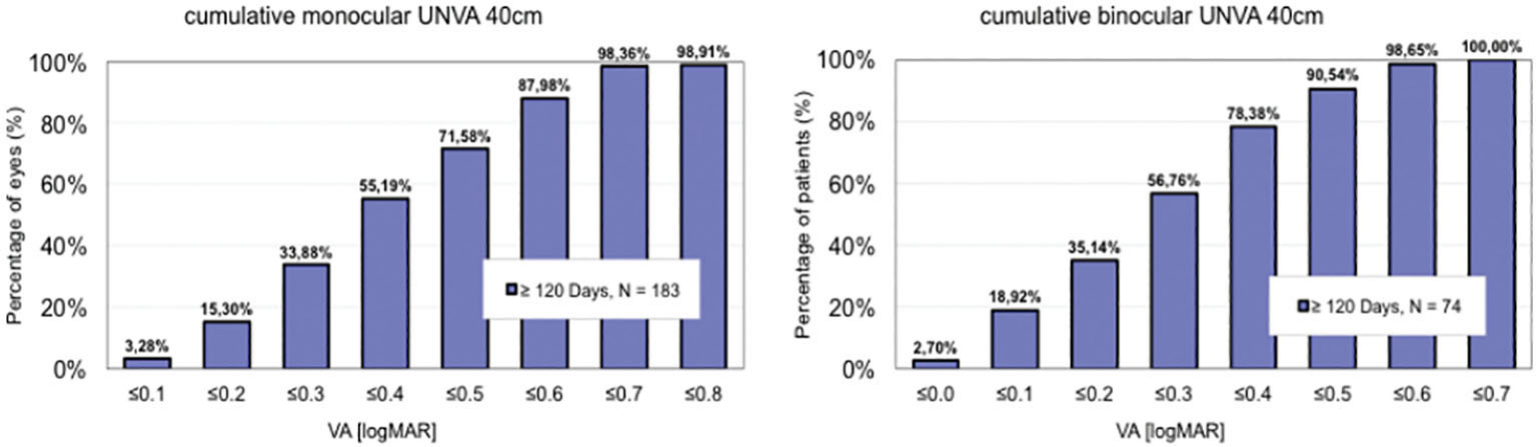
Near vision achieved.

**Figures 2** and **3** summarise the near vision achieved (4, 7).

**Figure 4:**
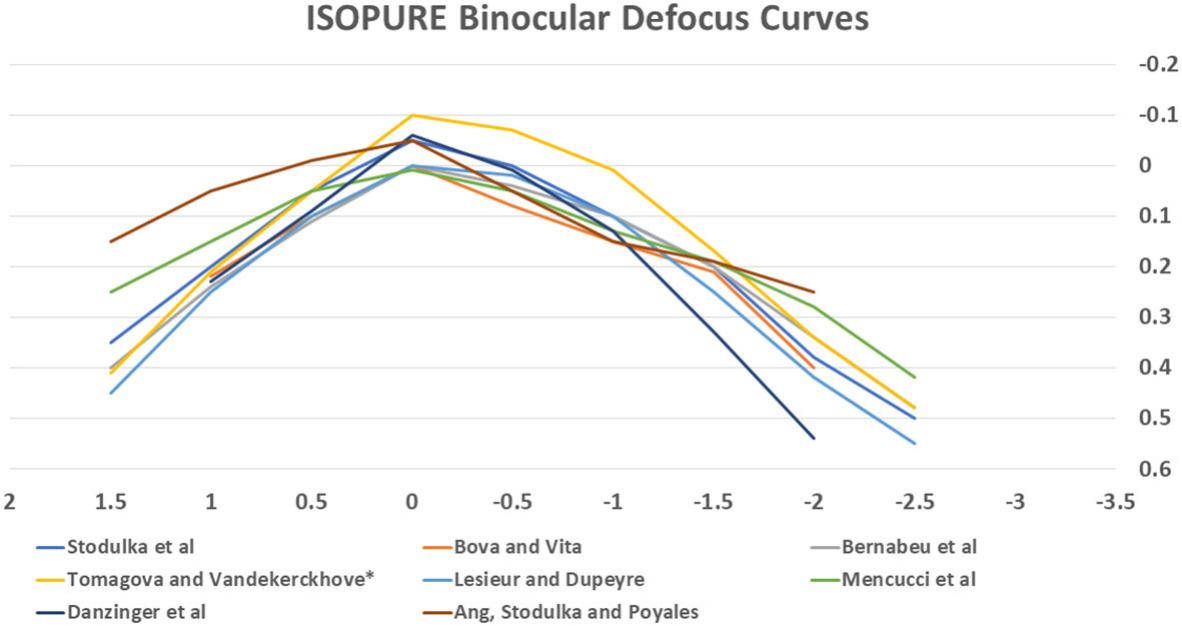
Defocus curves for BVI ISOPURE 123 IOL across the studies.

**Figure 4** summarises the defocus curves for ISOPURE 123 across the studies (N.B. the highest curve is a study with mini-monovision).

## Discussion

The ISOPURE^®^ IOL, which has an isofocal optic design, provides patients with a high degree of spectacle independence following cataract surgery. It provides excellent uncorrected distance vision and, in addition, provides good functional intermediate vision. With a mini-monovision refractive aim, useful near vision without spectacles is also achieved. Furthermore, there is no significant increase in the frequency of optical aberrations or dysphotopsias, meaning excellent quality of vision too.

Although currently there is good evidence for its visual performance, more studies are required with a greater number of participants in order to establish its true worth in providing high levels of patient satisfaction for spectacle independence and quality of vision.

Amongst the wide range of similar IOLs available currently, ISOPURE represents an excellent choice for surgeons and patients who seek outstanding functional vision and quality of vision with minimal spectacle dependence and photic phenomena. In addition, there is tolerance to residual astigmatism that is similar to that of a monofocal lens with pupil size up to 3.5 mm (11). A toric version of the IOL is awaited, as well as more data on posterior capsule opacification and YAG laser capsulotomy rates, which will become evident with long-term studies.

## Author contributions

EA: Conceptualization, Funding acquisition, Investigation, Project administration, Writing – original draft, Writing – review & editing.
